# Spatial Distribution of a Large Herbivore Community at Waterholes: An Assessment of Its Stability over Years in Hwange National Park, Zimbabwe

**DOI:** 10.1371/journal.pone.0153639

**Published:** 2016-04-13

**Authors:** Simon Chamaillé-Jammes, Anaïs Charbonnel, Stéphane Dray, Hillary Madzikanda, Hervé Fritz

**Affiliations:** 1 Centre d’Ecologie Fonctionnelle et Evolutive, CNRS UMR 5175, 1919 route de Mende, 34294, Montpellier Cedex 5, France; 2 Zone Atelier Hwange CNRS – Hwange LTER, PO Box 62, Dete, Zimbabwe; 3 Laboratoire Biométrie et Biologie Evolutive, CNRS UMR 5558, Université Lyon 1, 43 bd du 11 Novembre 1918, 69622, Villeurbanne Cedex, France; 4 Zimbabwe Parks and Wildlife Management Authority, Scientific Services, PO Box CY 140 Causeway, Harare, Zimbabwe; U.S. Geological Survey, UNITED STATES

## Abstract

The spatial structuring of populations or communities is an important driver of their functioning and their influence on ecosystems. Identifying the (in)stability of the spatial structure of populations is a first step towards understanding the underlying causes of these structures. Here we studied the relative importance of spatial vs. interannual variability in explaining the patterns of abundance of a large herbivore community (8 species) at waterholes in Hwange National Park (Zimbabwe). We analyzed census data collected over 13 years using multivariate methods. Our results showed that variability in the census data was mostly explained by the spatial structure of the community, as some waterholes had consistently greater herbivore abundance than others. Some temporal variability probably linked to Park-scale migration dependent on annual rainfall was noticeable, however. Once this was accounted for, little temporal variability remained to be explained, suggesting that other factors affecting herbivore abundance over time had a negligible effect at the scale of the study. The extent of spatial and temporal variability in census data was also measured for each species. This study could help in projecting the consequences of surface water management, and more generally presents a methodological framework to simultaneously address the relative importance of spatial vs. temporal effects in driving the distribution of organisms across landscapes.

## Introduction

Organisms are heterogeneously distributed in space. Understanding how abiotic (e.g. climate) and biotic (e.g. competition, predation) factors and their interactions across scales lead to the distribution patterns is a central goal of ecology. Unsurprisingly, there are thousands of descriptions of the spatial distribution of organisms, from within-populations [1] to species ranges [2] levels. What remains less understood is how these distributions vary over time. Species ranges determined by climatic factors appear mostly stable over decades [3]. At the population-scale, gradual demographic changes in density are generally associated with changes in habitat selection processes and distribution [4]. Extreme climatic events, on the opposite, can lead to immediate temporary redistribution of individuals [5]. Although these processes may be well understood in a few ecosystems, in most identifying the (in)stability of the distribution of individuals in a population is a first step towards understanding its underlying factors.

The stability of the distribution of individuals also has implications beyond basic ecology and may prove to be critical for ecosystem management. Indeed, the temporal consistency of the distribution allows managers to assess *a priori* what share of the population will be directly affected by some spatially-defined management practices (although a larger share could be indirectly affected by the potential redistribution of organisms). Such a situation—when a distribution is stable over time—should ease and strengthen the predictions of the consequences of management actions.

Surface-water management in semi-arid regions is one context where this could prove valuable. Surface-water provision is a widely used management practice in semi-arid grazing lands as increased water availability lead to an increase in livestock densities [6]. A similar approach has been developed during the 20^th^ century in many African protected areas to increase the abundance of wild large herbivores or sustain populations in ecosystems with limited or no permanent water [7,8,9,10]. Such increase in herbivore pressure often led to drastic changes in the vegetation around waterholes (“piosphere effect” [11]) and a uniform waterhole distribution could potentially lead to landscape homogenization. Artificial waterholes may also affect predator-prey relationships [12,13]. For these reasons traditional surface-water provision policies have been recently criticized [8,14,15,16]. Alternative policies have been discussed, with a strict no-artificial water policy being often considered unattractive by managers and possibly impairing conservation of water-dependent species due to the location of many protected areas in dry areas [15]. A more dynamic approach (based on regular opening/closure of artificial waterholes) aiming at maintaining minimum water availability but also spatial and temporal heterogeneity may be more suitable to simultaneously achieve objectives of large herbivore and landscape conservation [16]. The choice of artificial waterholes to be opened or closed every year however remains problematic and the impact of management actions would be easier to predict if the distribution of the herbivore communities at waterholes were stable over time.

Here we investigated the stability of the distribution of a large herbivore community drinking at waterholes in a semi-arid protected area, Hwange National Park (Zimbabwe). We used multivariate methods to study the spatio-temporal variations in abundance and composition of this community. Such a community-based approach (compared to a species-based one) makes it possible to address the natural scale of the ecosystem response to the management of water sources, in which waterhole openings/closures affect all species simultaneously.

## Methods

### Ethics statement

The authorization to conduct this research in the field was granted annually by the Zimbabwe Parks and Wildlife Management Authority. According to Zimbabwean and French laws no further ethical approval by a committee is required for such non-invasive, observational, study.

### Study area

**Fig 1:**
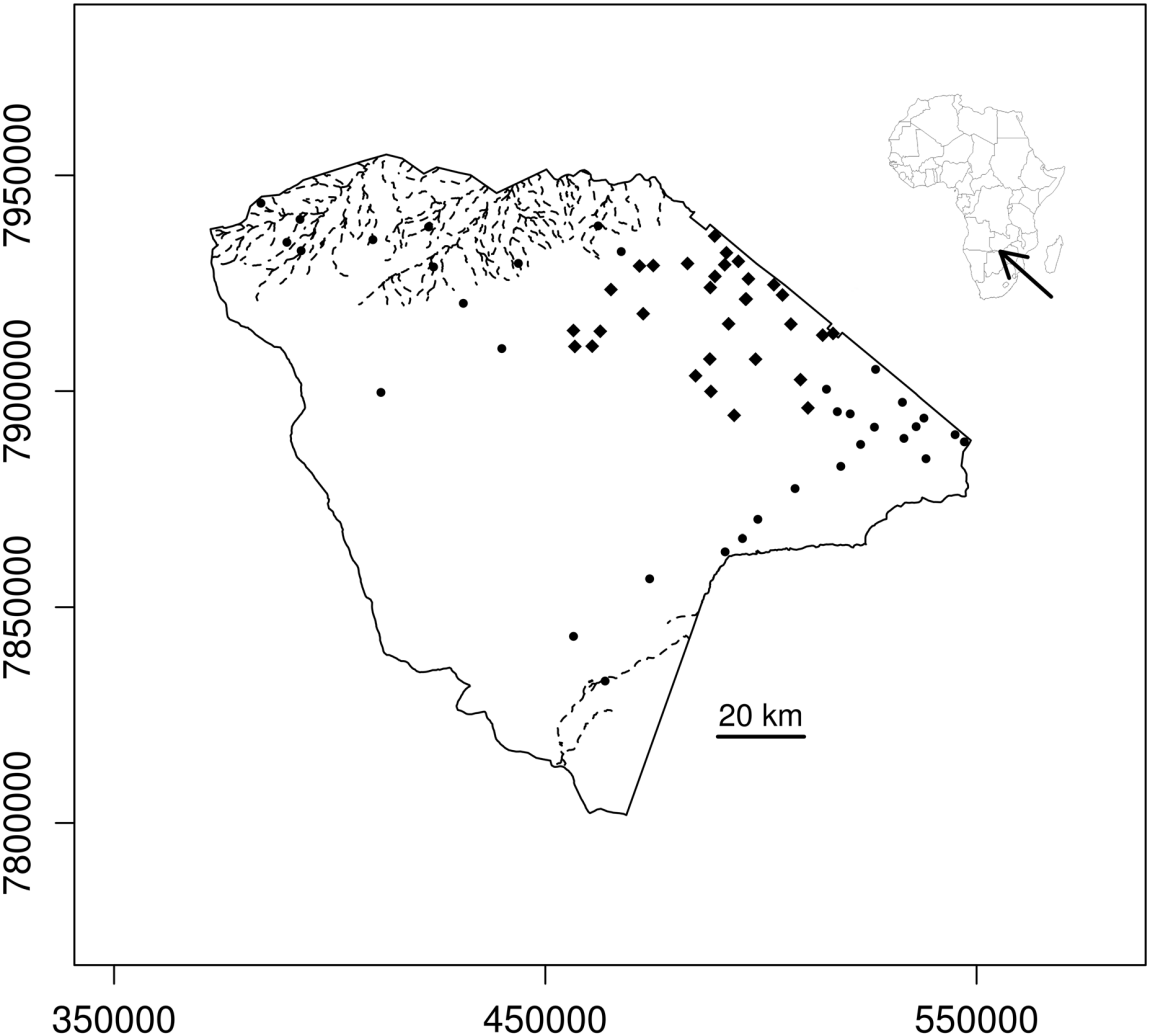
Map of the study area. The figure shows the locations of the waterholes surveyed (diamonds), and of artificial waterholes located outside the studied area (circles). Although waterhole size could vary from year to year, they were generally < 50 m in diameter at the time of the surveys. Rivers (dotted lines) dry up during the dry season, with only a few pools remaining. The solid line indicates the boundary of the Park.

Hwange National Park (hereafter HNP) covers c. 15000 km^2^ of dystrophic wooded savannas at the north-western border of Zimbabwe [17]. The park, neighboured by communal lands, forestry or safari areas, is unfenced and animals can move freely in and out. Rainfall mostly occurs between October and April. Rainfall data collected at the Main Camp station (N-18.0730°, E-26.9518°), and used here, show that annual rainfall averaged 606 mm on the long-run, with a coefficient of inter-annual variation of 25%. Thousands of natural pans hold rainfall water during the wet season. These natural pans dry up during the course of the dry season [10], with virtually all natural pans being dry by August during dry years (pers. obs.). Similarly, only a few pools remain in the river network at the end of the dry season. Artificial waterholes provide permanent water through pumping of underground water. Boreholes are unevenly distributed across the park, most being in located in the north-eastern region of the park (Fig 1). Pumping usually starts at the beginning of the dry season and is discontinued after the first significant rains. For more information about the surface-water dynamics of the park, see [10].

Here we focused on the Main Camp section (c. 1800 km). This section, located in the north-eastern region of the park (Fig 1), has more artificial waterholes than the centre of the park and therefore show large aggregation of herbivores in the dry season [18]. Although data are lacking, field experience suggests that there is not a strong rainfall gradient within this section.

This section also differs from the other sections by the vegetation and the associated herbivore community [18] making it an independent management unit. In this section, long-term herbivore densities of common species have been estimated, using road censuses, at: elephant (*Loxodonta africana*): ~ 4.36 per km^2^, giraffe (*Giraffa camelopardalis*): 1.47 per km^2^, impala (*Aepyceros melampus*): 1.43 per km^2^, kudu (*Tragelaphus strepsiceros*): 1.59 per km^2^, roan antelope (*Hippotragus equinus*): 0.05 per km^2^, sable antelope (*Hippotragus niger*): 0.22 per km^2^, blue wildebeest (*Connochaetes taurinus*): 0.79 per km^2^, Burchell’s zebra (*Equus quagga*): 1.15 per km^2^). For more details see [18].

### Herbivore data

We used data on the abundance of herbivore at waterholes, collected once a year during the hot dry season (late September—early October) at the artificial and the few remaining natural waterholes. Twenty-four hours counts were conducted at full moon, from midday, and the number of animals coming to drink was recorded for all species. Here we analyzed data for the 8 most common large herbivore species: elephant, giraffe, greater kudu, impala, roan antelope, sable antelope, blue wildebeest and Burchell’s zebra. Data are provided in S1 File.

The park is characterized by a historically high elephant density. Culling operations maintained elephant density around 1 per km^2^ until 1986 when they were discontinued. Density then increased quickly until 1992 and has been fluctuating since then [19]. Changes in the spatial distribution of elephants associated with these changes in density have been described elsewhere [19,20]. In order to avoid that these known changes in elephant densities affect our analysis we used data collected from 1992 to 2005.

Data from 1997 were not included in the analyses because rains occurred during the survey, and animals were able to drink in the numerous ponds created. Contrary to other years, the number of animals counted in 1997 was then very low and not representative of dry season abundances at waterholes.

These counts are total counts and have no estimation of uncertainty associated to them. Bias or imprecision might exist: for instance, numbers could be affected by observer effects, or the size of the largest groups of herbivores could have been underestimated. These effects have not yet been quantified, although these are likely to be small thanks to the good visibility at waterholes and the generally small size of groups. In the context of the current analysis, we assumed that no spatial or temporal patterns would exist in these effects, and thus that they would not have biased our analyses.

### Statistical analyses

We studied the spatial organization of the herbivore community and its stability over time using multivariate analyses. The original data table contained the abundances of the 8 species (columns) for 31 waterholes sampled during 13 years. This corresponded only to 259 censuses (rows) because waterholes that were dry for a given year (i.e. no water and thus no herbivores) were excluded from the dataset. Data were standardized by species (i.e. abundance at waterholes was divided by species-level standard deviation) because elephants, being at least ten times more abundant than other species, would otherwise have had an overwhelming influence on the analyses. Moreover, we did not center the data to focus on variations in abundance relative to the absence of species, rather than relative to their long-term mean abundances. For details on the implications of data transformation (standardization and centering) in multivariate analysis, the reader could consult [21].

We used a principal component analysis (hereafter PCA) to identify the main variations in the community composition. This general analysis aggregates both the spatial and temporal variability of the dataset. The length of the gradient was 2.13, which justified the use of PCA [22]. Moran’s I showed no evidence of spatial autocorrelation in the component scores (not shown). We introduced the information relative to the sampling by using within- and between-group PCAs [23], with group referring here to either waterholes (spatial grouping) or years (temporal grouping). If we consider the spatial grouping, it is possible to decompose the total variability into two additive parts corresponding to spatial variations in herbivore abundance stable over time (differences between waterholes which are linked to the identity of waterholes) and non-spatial variations. Between-waterhole PCA emphasizes the differences between waterholes (spatial variation) by identifying the species for which abundance vary the most between waterholes. A randomization test can be conducted to test if the differences among waterholes are significant or not [24]. On the other hand, within-waterhole PCA removes the waterhole effect (by centering the data by waterhole) and thus identifies the main structures that are not explained by the differences among waterholes (non spatial variation), and can reveal temporal variability. Similar reasoning can be applied for years in between- and within-year PCAs.

We investigated how the total variance could be decomposed across spatial and temporal effects using between- and within-waterhole PCAs and between- and within-year PCAs. Between-waterhole and between-year PCAs directly estimated the contribution to the variability of waterholes and years respectively. Within-waterhole and within-year PCAs on the contrary estimated the share of variance not explained by waterhole and years respectively. Comparison of within-year and between-waterhole PCAs thus allowed assessing how much of the spatial variability (i.e. variability not explained by years: within-year PCA) was explained by waterhole identity (between-waterhole PCA).

We investigated if the spatial variability of herbivore abundance at waterholes was related to the distribution of the vegetation around these waterholes. We did so by using a published Landsat-based vegetation map which distinguished 6 vegetation types representing increasing woody cover (grassland, three types of bushland representing a gradient of openness, wooded bushland, woodland)(for more details see [13]).

For each waterhole, we calculated the proportion of each vegetation type within an 8 km radius. This distance was chosen arbitrarily, but results were qualitatively similar if calculated over 5 or 10 km. We then performed a redundancy analysis [25], using the table of herbivore abundance as response table, and the table of the proportion of each vegetation type at waterholes as explanatory table. This allowed us to compute the proportion of the total and spatial (i.e. between-waterhole) variation in herbivore abundance at waterholes associated with the distribution of the vegetation.

All analyses were conducted with the ade4 package of the R software [26].

## Results

### General PCA

**Fig 2:**
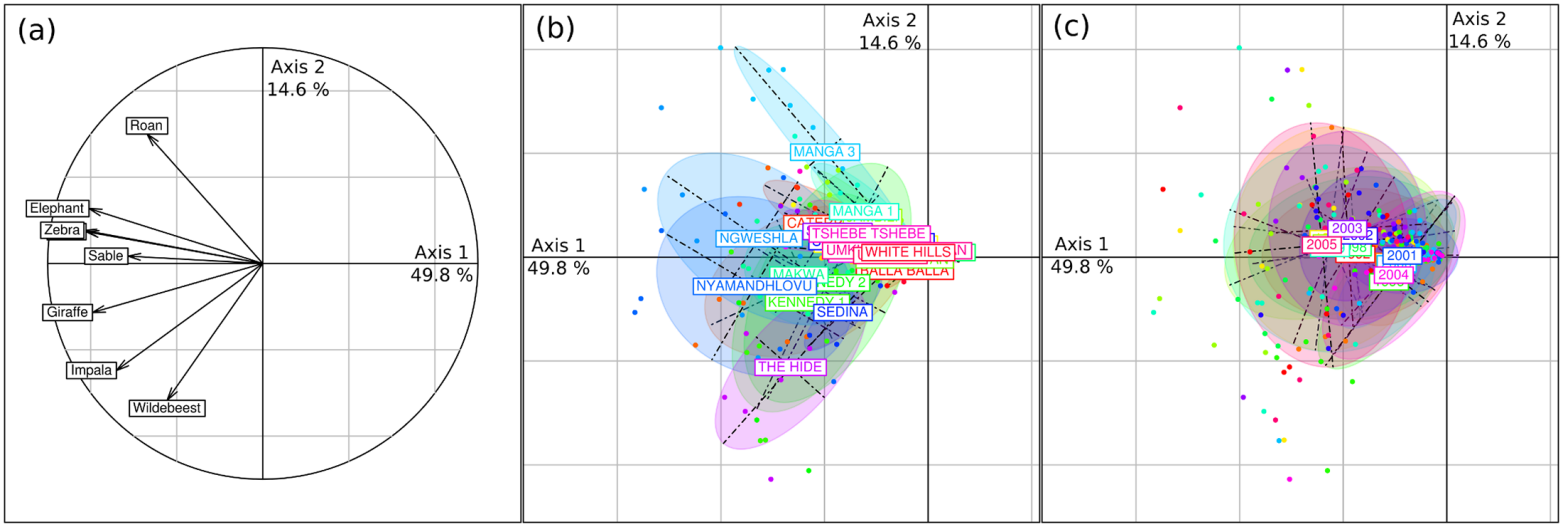
Results of the general principal component analysis. Correlations of individual variables (species abundance at waterholes) with axes 1 and 2 are shown in (a). Scatterplots of the PCA results on the first two axes are shown in (b,c). Each census (black dot) is linked to the centroid of all censuses conducted (b) at the same waterhole or (c) during the same year—an ellipse encompass the closer 66% of all censuses conducted (b) at the same waterhole or (c) during the same year.

The general PCA allowed representing an important part of the total variability of the data, as together axis 1 and 2 overall explained 64.4% of the variability of the data. The first axis represented a gradient of abundance at waterholes for most species and census data were well spread over this axis (Fig 2).

The second axis separated censuses along a species composition gradient, mainly driven by abundance at waterholes of roan, wildebeest and impala (Fig 2a). The comparison of the amount of variation explained by axis 1 (49.8%) and 2 (14.6%) revealed that most of the variability between the censuses was linked to a global variation of herbivore abundance rather than variation between species composition. Additionally, although this analysis did not allow distinguishing between spatial and temporal variability, representing waterholes or years on the PCA scatterplot suggested that more variability was accounted for by spatial (i.e. waterholes) than by temporal (i.e. year) effects (compare Fig 2b and 2c, ellipses appear generally larger in Fig 2c). This was confirmed by between- and within-group PCAs.

### Spatial variability and its stability: within-year and between-waterhole PCAs

**Fig 3:**
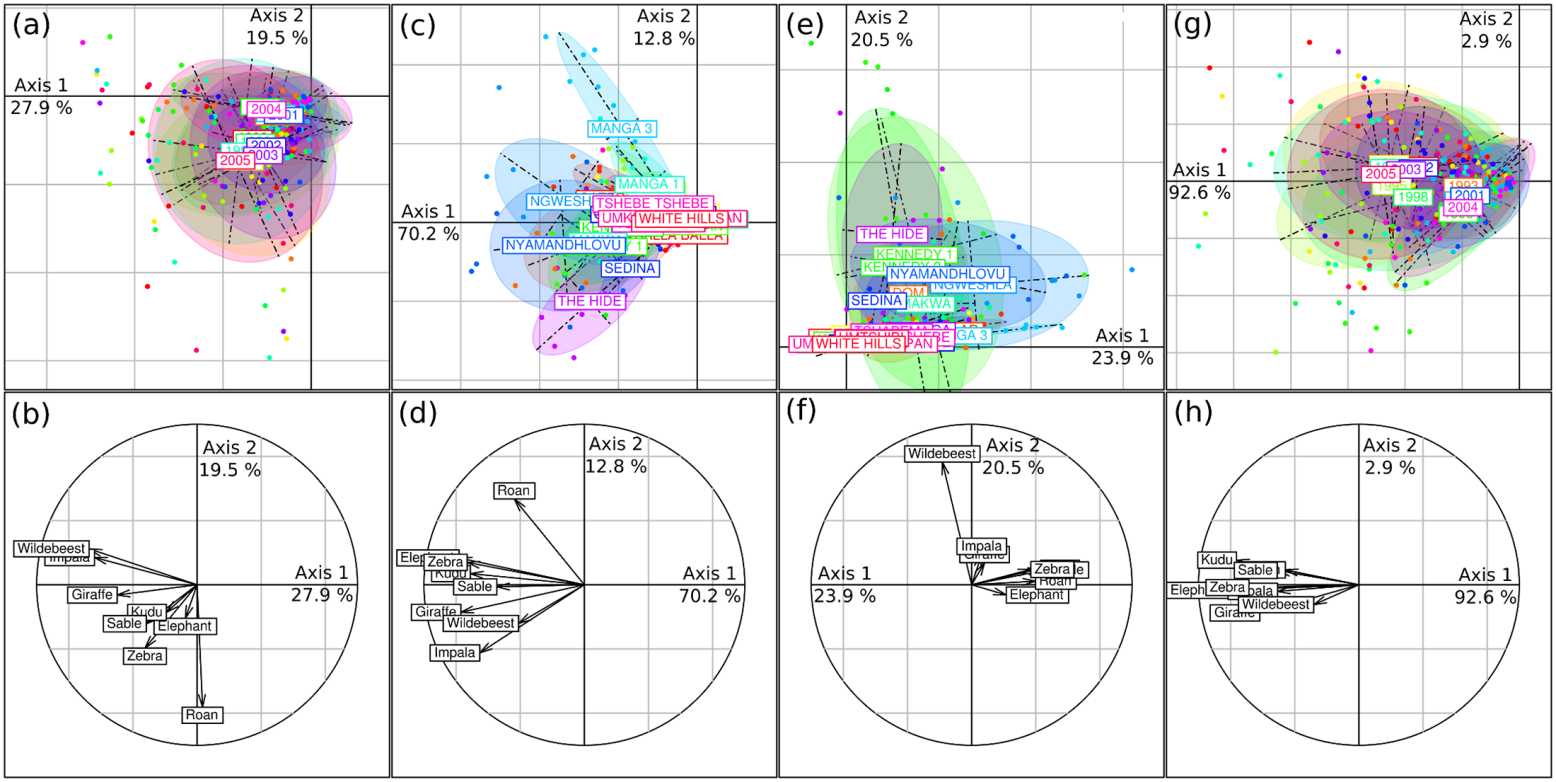
Results of the within-year (a,b), between-waterhole (c,d), within-waterhole (e,f) and between-year (g,h) principal component analyses. Scatterplots of their results on the first two axes are shown in (a,c,e,g). Each census (black dot) is linked to the centroid of all censuses conducted (c,e) at the same waterhole or (a,g) during the same year—an ellipse encompass the closer 66% of all censuses conducted (c,e) at the same waterhole or (a,g) during the same year. Correlations of individual variables (species abundance at waterholes) with axes 1 and 2 are shown in (b,d,f,h).

**Fig 4:**
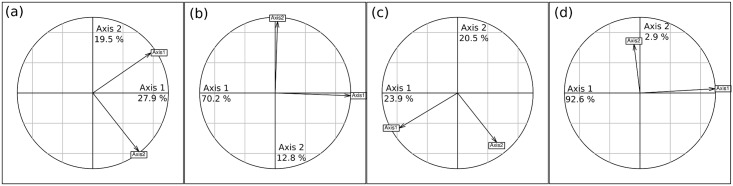
PCA comparisons. Projections of axes 1 and 2 (arrows) of the general principal component analysis (PCA) onto the first two axes of the (a) within-year, (b) between-waterhole, (c) within-waterhole, (d) between-year PCAs.

The within-year PCA (Fig 3a) removed the year effect and showed that 61.0% of the total variability was due to intra-annual (i.e. spatial) variability. An important part of the variability between censuses could thus possibly be related to spatial variability in species composition and abundance. However the within-year PCA did not find a strong structure within this variability, as axis 1 and 2 represented only 27.9 and 19.5% of the intra-annual variation respectively. These two axes separated censuses on the basis of species composition, with waterholes being separated by wildebeest, impala or giraffe abundance along axis 1 and roan and other species along axis 2 (Fig 3b—see also Fig 4a for a comparison with the general PCA).

The between-waterhole PCA (Fig 3c) also explained an important part of the total variation (59.1%; permutation test: P < 0.001), which was mainly associated to the first axis (70.2% for axis 1 and 12.8% for axis 2). Thus, independently of the year, waterholes could often be differentiated by herbivore abundance (axis 1; Fig 3d) and to a lesser extent by abundance of species well represented along axis 2 (roan, wildebeest, impala; Fig 3d). Representation of species along axes 1 and 2 was similar to their representation along axis 1 and 2 of the general PCA (Fig 4b), confirming that between-waterhole variability represented the main source of variability. The redundancy analysis revealed that 40.1% and 67.8% of the total and spatial (i.e. between-waterhole) variability in herbivore abundance, respectively, was associated with the distribution of the vegetation. Altogether these results suggested that consistent differences in herbivore abundance between waterholes, well associated with vegetation distribution, were a dominant aspect of these long-term data.

### Temporal variability: within-waterhole and between-year PCAs

The within-waterhole PCA (Fig 3e) removed the waterhole effect and explained 40.9% of the variation. This variability was however not well structured (only 23.9 and 20.5% of the within-waterhole variation is associated to axis 1 and 2 respectively), and the correlation between the species abundance and these axes differed from the general PCA (Figs 3f and 4c).

**Fig 5:**
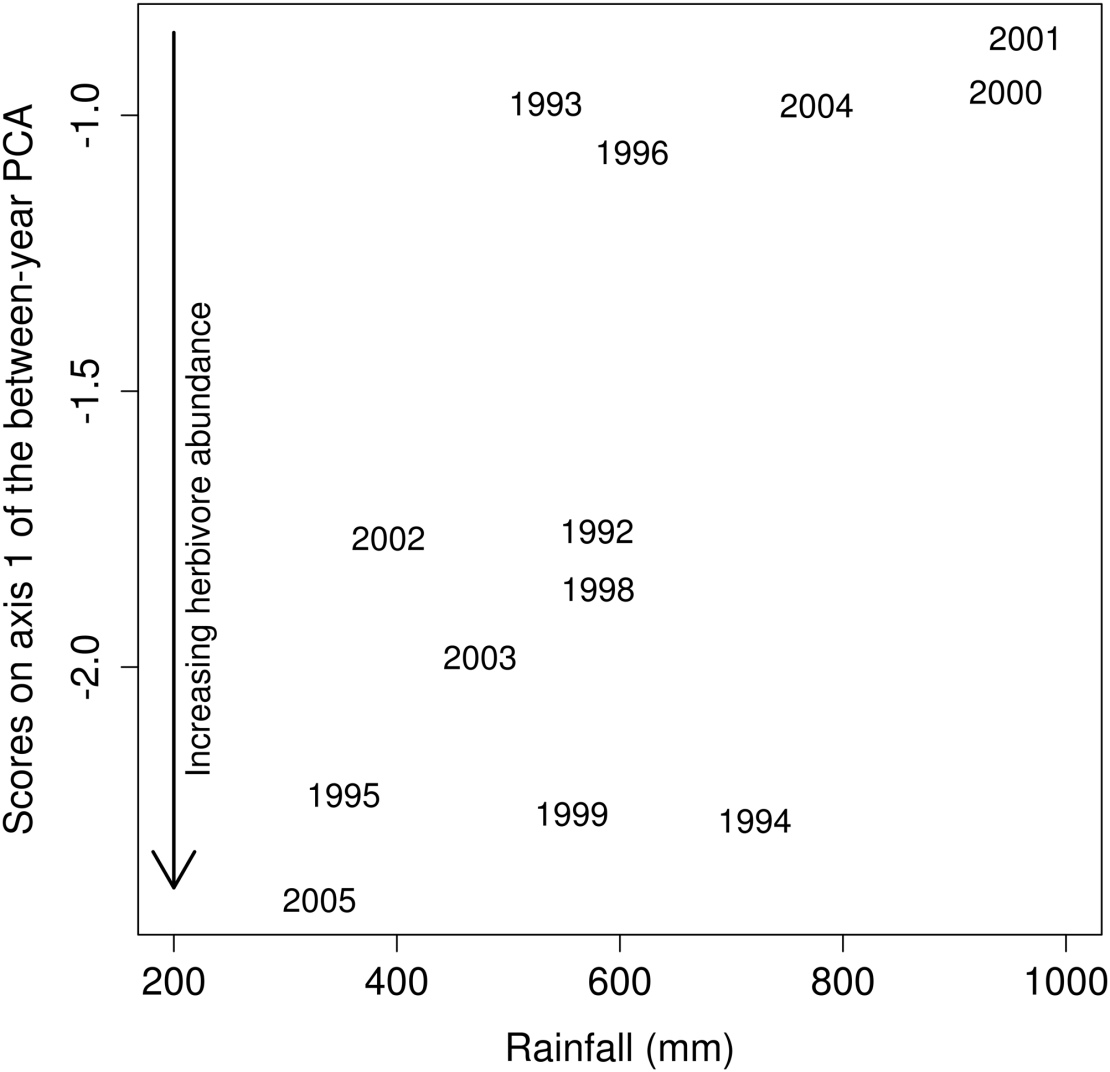
Effect of annual rainfall on herbivore abundance at waterholes. Relationship between annual rainfall and component scores from the first axis of the between-year principal component analysis (Pearson r = 0.68, P = 0.01). This first axis represented 92.6% of the inter-annual variation in herbivore abundance at waterholes, and lower values indicated lower herbivore abundance.

The between-year PCA (Fig 3g) explained only 39.0% of the total variance (permutation test: P < 0.001). This moderate but significant inter-annual variability in the census data was almost completely represented on the first axis of the between-year PCA (92.6% of the inter-annual variation). This axis was very similar to axis 1 of the general PCA and described a gradient of increasing abundance of all herbivores at waterholes (Figs 3g and 4d). Scores of the years along this axis were well correlated with rainfall of the year (Fig 5; Pearson r = 0.68, P = 0.01), indicating that global herbivore abundance at waterholes increased during dry years. The second axis explained virtually none of the variation (2.9%).

### Species differences

Table 1 summarizes, for individual species, the amount of variance in species abundance explained by inter-annual variability (estimated from the between-year PCA) and spatial variability linked to waterhole identity (estimated from between-waterhole PCA). Elephant and kudu abundance at waterholes varied between years and waterholes to a similar extent. For other species, abundance varied much more between waterholes than between years, although this was slightly less marked for zebras whose abundance at waterholes still varied moderately between years.

**Table 1:** Amount of variance (%) in species abundance at waterholes explained by temporal (between-year) or spatial (between-waterhole) variations, obtained from between-year and between-waterhole PCA respectively. See text for details.

Species	Year	Waterhole
Elephant	29.5	26.8
Giraffe	4.8	55.9
Impala	4.9	59.6
Kudu	26.8	32.1
Roan	7.2	48.2
Sable	7.3	26.1
Wildebeest	2.2	28.5
Zebra	11.8	36.1

## Discussion

### Spatial vs. temporal effects in community variability at waterholes

We used a unique dataset of herbivore census data collected over more than 10 years to investigate the relative importance of spatial and temporal variability of herbivores abundance at waterholes. Using a statistical approach focused on community-level variability, our results showed (1) that variability in herbivore abundance (rather than composition) between censuses explained an important part of the total variability of the dataset (see the general PCA), (2) that this variability was mainly due to some differences between waterholes which were stable over time. Indeed, the between-waterhole PCA explained an important part of the variance (59.1%), almost as much as the within-year PCA (61%) which estimates the amount of variability unexplained by interannual difference. Stated differently, in HNP some waterholes consistently display higher herbivore abundance than others during the dry season. Although data on waterhole attendance are scarce, some previous studies have showed that attendance may differ widely across waterholes [7,27,28]. Those studies were however all conducted over only one dry season. Our study thus brings a much-needed long-term perspective which raises questions about the causes underlying the stability of the herbivores distribution.

Our study showed that the spatial distribution of abundance of herbivore at waterholes was strongly associated with the distribution of the vegetation in the landscape. One could assume that habitat selection of herbivores drives this pattern, and explains the spatial stability in herbivore abundance at waterholes in HNP where vegetation structure did not display major changes over the study period [29]. This is thought to happen elsewhere: in Kruger National Park (South Africa) browsing animals may be less attracted to artificial waterholes than to river pools because of the riverine vegetation nearby [30]. However, we caution against accepting this interpretation without further studies in HNP. Herbivores impact vegetation, and variation in total herbivore abundance at waterholes (which is the main source of variation in the dataset, see general PCA), sustained in the long-term, is likely to induce contrasts in vegetation structure. Thus, waterholes with consistently higher herbivore pressure may be surrounded by different vegetation than those experiencing less pressure. The stability of the variation in herbivore abundance at waterholes may actually contribute to explain why gradients of vegetation impacts of herbivore around waterholes appear heterogeneous at the landscape scale in HNP [31]. Clearly, further studies, particularly studies of herbivore habitat selection, are required to solve the chicken-and-egg problem of the association between vegetation and herbivore abundance. In any case, we emphasize that to prevent homogenization of vegetation across landscape due to water provision managers should aim at maintaining both spatial heterogeneity in herbivore distribution and some levels of stability of this distribution. “Rotating” herbivore pressure using artificial waterholes, although maintaining spatially heterogeneous pressure in any year, might be detrimental on the long-term by spatially averaging herbivore pressure.

Although differences in herbivore abundance between waterholes were consistent across years, herbivore abundance generally increased during dry years. This is an unexpected pattern in semi-arid ecosystems where drought usually increases mortality. The pattern observed in this study could be linked to the distribution of surface-water availability across the park. As annual rainfall decreases, water availability during the dry season in the south-western regions of the park with little or no artificial waterhole decreases [10], and herbivores aggregate in sections of the park where artificial waterholes are numerous, as in the study area [18]. Therefore, inter-annual changes in herbivore abundance at waterholes observed here are more likely to represents migration than demographic processes. As changes in abundance associated with rainfall fluctuations largely dominated other year-to-year variations, our results highlight that at the scale of the study other factors affecting the temporal dynamics of herbivores (e.g. predators, poaching) were overwhelmed by the rainfall-driven redistribution of herbivores across the Park.

Relatively to the spatial variation in abundance between waterholes, inter-annual fluctuations in abundance were important only for elephants and kudu however. These species are among the most ubiquitous in HNP. They are known to be generalist browsers during the dry season when surveys were conducted [32]. They are also known to be highly mobile (elephants: [33], kudus: unpublished GPS data). Altogether, this may make them less constrained than other species in this bushy environment, and likely to be able to spatially and temporally adjust to water availability and habitat conditions. This also means that, as long as surface water is available, managing the spatial distribution of these species might be complex.

Finally, but importantly, the role played by water quality remains to be studied. Herbivores are known to respond to water quality to some extent [34]. In HNP it had been shown for instance that sodium concentration in water influenced the distribution elephants at low, but not at high, density, probably because competition for forage became a dominant driver of their distribution [35]. Monitoring water quality is however challenging, as many biophysical variables need to be studied, and some can change rapidly. It is probably for this reason that reports on long-term temporal dynamics of water quality in savanna ecosystems are rare. In HNP, this is made even more complex by the fact that groundwater pumping likely disrupts the ecological dynamics (e.g. eutrophization) occurring in natural ponds. Therefore, further studies are required to disentangle the respective role of water quality and habitat selection in driving herbivore abundance at waterholes.

### Perspectives for surface water management

Our results shed a new light on water management in protected areas and offer promising perspectives. The traditional practice of a static and spatially extended water provision is being questioned mostly on the basis of herbivore impact on vegetation, and alternative strategies are being discussed [8,14,15,16]. One approach could be a more dynamic practice of opening/closure of artificial waterholes [15]. Such policy is likely to be successful if (1) it is conducted within an adaptive management framework with for instance actions based on past assessment of vegetation status and herbivore abundance, (2) managers could reliably predict the distribution of herbivores under various management scenarios, and particularly under current management (i.e. no change policy). Our results partially supported point (2) by showing that herbivore distribution was quite stable across years and associated with vegetation distribution. The temporal variability appeared to be very well explained by annual rainfall, probably because it influenced the availability of surface water outside the studied area. As annual rainfall is known at the time the decision on which waterholes to pump has to be taken (early dry season), herbivore distribution at waterholes in the absence of waterhole closure is likely to be predictable to a reasonable extent, and managers could adjust their policy accordingly. However, this may hold only as long as herbivore densities do not change too much, due for instance to dramatic drought-induced mortality or poaching upsurge. In such situation, density-dependent habitat selection processes may induce unpredictable (based on past data) changes in the distribution of some species. Therefore, to test the generality of our results, we call for similar studies to be conducted elsewhere in places which may have experienced different dynamics. More generally, we emphasize that an investigation of the temporal dynamics of population distribution is an important tool for management-orientated landscape ecology.

## Supporting Information

S1 File Herbivore abundance data. (CSV)
